# Loss of autism-candidate CHD8 perturbs neural crest development and intestinal homeostatic balance

**DOI:** 10.26508/lsa.202201456

**Published:** 2022-11-14

**Authors:** Gaëlle Hayot, Mathieu Massonot, Céline Keime, Elodie Faure, Christelle Golzio

**Affiliations:** 1 Institut de Génétique et de Biologie Moléculaire et Cellulaire, Illkirch, France; 2 Centre National de la Recherche Scientifique, Illkirch, France; 3 Institut National de la Santé et de la Recherche Médicale, Illkirch, France; 4 Université de Strasbourg, Strasbourg, France

## Abstract

Individuals with mutations in *CHD8* present with gastrointestinal complaints, yet the underlying mechanisms are understudied. Here, using a stable constitutive *chd8* mutant zebrafish model, we found that the loss of *chd8* leads to a reduced number of vagal neural crest cells (NCCs), enteric neural and glial progenitors, emigrating from the neural tube, and that their early migration capability was altered. At later stages, although the intestinal colonization by NCCs was complete, we found the decreased numbers of both serotonin-producing enterochromaffin cells and NCC-derived serotonergic neurons, suggesting an intestinal hyposerotonemia in the absence of *chd8*. Furthermore, transcriptomic analyses revealed an altered expression of key receptors and enzymes in serotonin and acetylcholine signaling pathways. The tissue examination of *chd8* mutants revealed a thinner intestinal epithelium accompanied by an accumulation of neutrophils and the decreased numbers of goblet cells and eosinophils. Last, single-cell sequencing of whole intestines showed a global disruption of the immune balance with a perturbed expression of inflammatory interleukins and changes in immune cell clusters. Our findings propose a causal developmental link between *chd8*, NCC development, intestinal homeostasis, and autism-associated gastrointestinal complaints.

## Abstract (precis)

A combination of zebrafish and transcriptomic analyses revealed the role of the autism-candidate chd8 in the development of the enteric nervous system, serotonin metabolism, and intestinal integrity.

## Introduction

Autism spectrum disorders (ASD) are a group of heterogeneous diseases, characterized by two core symptoms: difficulties in social communication and interactions; and restricted, repetitive, and stereotyped behavior and interests. In more than 80% of cases, ASD is associated with one or several comorbidities, including intellectual disability, head circumference defects (i.e., micro/macrocephaly), facial phenotype, attention-deficit/hyperactivity disorder, marked sleep dysfunction, and increased rates of gastrointestinal (GI) complaints (constipation, diarrhea, abdominal pain, and/or bloating) (Levy et al, 2010).The prevalence of the GI symptoms in autism varies greatly depending on data collection and methodological approaches: reports indicate rates ranging from 4.2% to 96.8% (Buie et al, 2010; Mazurek et al, 2013; Holingue et al, 2018). Despite the increasing awareness of the GI complaints in ASD and their impact on the quality of life of the patients and their family, the etiology of these ASD-associated endophenotypes has not been thoroughly studied.

Here, to tackle this challenge, we took advantage of the strong association between mutations in the autism-candidate *CHD8* (chromodomain helicase DNA-binding protein 8; MIM*610528) and GI complaints. *CHD8* is one of the most frequently found mutated genes in ASD cases (0.21% of individuals presenting with ASD) (Neale et al, 2012; O’Roak et al, 2012; Sanders et al, 2012; Bernier et al, 2014; Ostrowski et al, 2019; Siu et al, 2019; An et al, 2020). Heterozygous loss-of-function mutations in *CHD8* define an ASD subtype (MIM#615032) with 80% of *CHD8* cases presenting with GI complaints, of which a total of 60% have recurring periods of considerable constipation followed by loose stool or diarrhea (Bernier et al, 2014; Douzgou et al, 2019). We have previously shown that the transient knockdown of *chd8*, the sole ortholog of *CHD8* in zebrafish exhibiting a high ubiquitous expression from two-cell stage to five-somite stage in the embryo and then restricted expression in the brain and intestinal tract from 3 days post-fertilization (dpf) onward, leads to a reduced number of enteric neurons and compromised intestinal motility, which is consistent with the constipation periods reported by individuals carrying *CHD8* truncating mutations (Bernier et al, 2014). However, it remains unclear how *chd8* acts during the development of the enteric nervous system (ENS) and whether *CHD8*-associated GI complaints are solely due to impaired neuronal function in the intestine.

All enteric neurons and glia are neural crest cell (NCC) derivatives (Burns et al, 2002). The development of the ENS is conserved between humans and zebrafish, although it is simplified in the latter (Fu et al, 2004; Olden et al, 2008). In humans, the ENS derives from the vagal and sacral NCCs (Fu et al, 2004). In humans, mice, and chicken, vagal NCCs provide most of the enteric progenitors that colonize the entire length of the digestive tract, whereas sacral NCCs generate a small number of enteric progenitors that colonize exclusively the posterior intestine (Burns & Le Douarin, 2001). In zebrafish, the sacral neural crest has never been described and the ENS derives mainly from the vagal neural crest (Olden et al, 2008) and from the Schwann cell precursors deriving from the trunk neural crest (El-Nachef & Bronner, 2020). After leaving the dorsal part of the neural tube, around 24 hpf, vagal NCCs migrate to the intestine and enter it at ∼32 hpf. Then, they migrate, in two parallel lines, from the anterior region of the intestine to its posterior extremity, which they reach at 66 hpf. In the meantime, enteric neuronal progenitors undergo proliferation and start to differentiate as early as 54 hpf to form a functional ENS by 5 dpf (Olden et al, 2008). Here, we combined zebrafish phenotypic analyses and transcriptomic approaches to examine these key developmental processes.

In addition to a fully functional ENS, a healthy gut possesses an efficient intestinal mucosal barrier that ensures an adequate containment of undesirable non-sterile contents present within the intestinal lumen. When the mucosal barrier is compromised, micro-organisms and dietary antigens trigger the innate immune response. In inflammatory bowel diseases (IBD) such as ulcerative colitis and Crohn’s disease, the immune system responds inappropriately to environmental triggers, which causes chronic intestinal inflammation (Brandwein et al, 1997; Matsumoto et al, 1998; Abraham & Cho, 2009). Individuals with IBD suffer from abdominal pain and impaired GI transit (Sairenji et al, 2017), which are reminiscent of *CHD8*-associated GI complaints. We thus sought to determine whether the intestinal homeostasis could be affected by *chd8* heterozygous loss.

In this study, we used a stable constitutive *chd8* mutant zebrafish line to model the GI disturbances associated with ASD and we determined the consequences of a heterozygous loss of *chd8* on the development of the ENS and the intestinal homeostasis in larval and adult stages. First, we found that the heterozygous loss of *chd8* leads to a reduced number of vagal NCCs emigrating from the neural tube at 24 hours post-fertilization (hpf). Their early migration capability was altered at 48 hpf. At 5 dpf, the intestinal colonization is complete in *chd8* mutants, but the NCC differentiation is perturbed with a decreased number of NCC-derived serotonergic neurons. In addition, we found that the number of serotonin-producing enterochromaffin cells is reduced, suggesting a hyposerotonemia in the intestine of *chd8* mutants. These observations are further confirmed by transcriptomic analyses of NCC-derived neurons that showed an altered expression of key receptors and enzymes in serotonin and acetylcholine signaling pathways. Second, we determined that the intestinal architecture, itself, is compromised in the absence of *chd8*. We observed a thinner intestinal epithelium accompanied by an accumulation of neutrophils and the decreased numbers of goblet cells and eosinophils in the adult intestine, suggesting that the mucosal barrier is compromised when *chd8* is absent. Last, single-cell sequencing of the whole intestine showed a global disruption of the immune balance in *chd8* mutants with a perturbed expression of inflammatory interleukins, changes in immune cell clusters, and active pro-inflammatory immune response. Taking our data together, we propose a causal developmental link between *chd8*, impairment of the NCC development, dysregulation of the serotonergic pathway, alterations of the intestinal and immune homeostasis, and autism-associated GI complaints.

## Results

### Phenotypic characterization of stable zebrafish mutant line *chd8* ^*sa19827*^

**Figure S1.**
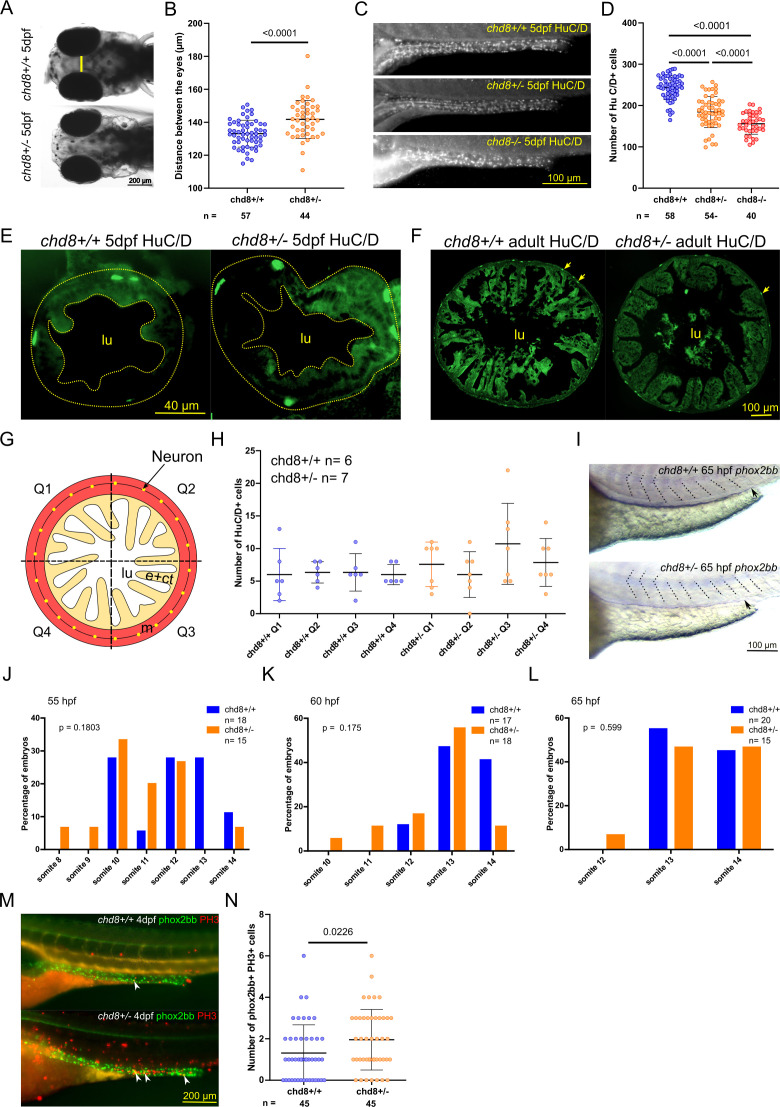
Zebrafish mutant line *chd8* ^*sa19827*^ exhibited the macrocephaly, loss of enteric neurons, increased neural crest cell (NCC) proliferation, but no NCC migration defect between 55 and 65 hpf. **(A)** Representative dorsal images of *chd8*^*+/+*^ and *chd8* ^*sa19827/+*^ zebrafish larvae at 5 days post-fertilization (dpf). The yellow line shows how the head was measured. **(B)** Dot plot of the measured head size for each condition tested. Welch’s *t* test was conducted between pairs of conditions. **(C)** Representative lateral images of the mid- and posterior intestines of *chd8*^*+/+*^, *chd8*^*sa19827/+*^, and *chd8*^*sa19827/sa19827*^ zebrafish larvae at 5 dpf stained with anti-HuC/D monoclonal antibody to visualize the enteric post-mitotic neurons. **(D)** Dot plot of HuC/D-positive cells for each condition tested. A *t* test was conducted between pairs of conditions. **(E)** Representative images of intestinal cross sections of *chd8*^*+/+*^ and *chd8* ^*sa19827/+*^ zebrafish larvae at 5 dpf that underwent immunostaining against HuC/D. The limit between the lumen and the epithelium and the border of the muscle layers are depicted by dashed lines. **(F)** Representative images of intestinal cross sections of *chd8*^*+/+*^ and *chd8* ^*sa19827/+*^ adult zebrafish that underwent immunostaining against HuC/D. HuC/D-positive cells are shown by yellow arrows. **(G)** Schematic showing the four intestinal quadrants defined to quantify the HuC/D-positive cells. **(G, H)** Dot plot showing the number of HuC/D-positive cells in each of the four quadrants shown in (G), for each condition tested. A Kruskal–Wallis test was performed. **(I)** Representative lateral images of *chd8*^*+/+*^ and *chd8*^*sa19827/+*^ zebrafish larvae at 65 hpf. In situ hybridization was performed using a probe against *phox2bb*. The position of the front of migration is shown by a black arrow. **(J)** Histogram of the position of the front of migration of enteric NCCs at 55 hpf, using somites as morphological landmarks. Fisher’s exact test was conducted between pairs of conditions. **(K)** Histogram of the position of the front of migration of enteric NCCs at 60 hpf, using somites as morphological landmarks. Fisher’s exact test was conducted between pairs of conditions. **(L)** Histogram of the position of the front of migration of enteric NCCs at 65 hpf, using somites as morphological landmarks. Fisher’s exact test was conducted between pairs of conditions. **(M)** Representative lateral images of the intestine of *Tg2*(*phox2bb:E*GFP);*chd8*^*+/+*^ and *Tg2(phox2bb:EGFP);chd8*^*sa19827/+*^ zebrafish larvae at 4 dpf stained with anti-phospho-histone H3 (PH3) monoclonal antibody to visualize the proliferating cells. Double-positive phox2bb+/PH3+ cells are shown by white arrowheads. **(N)** Dot plot of the number of *phox2bb*-positive/PH3-positive cells for each condition tested. A Mann–Whitney test was conducted between pairs of conditions. lu, lumen; e, epithelium; ct, conjunctive tissue; m, muscle layers; and n, number of larvae or adult fish.

We obtained a zebrafish mutant line carrying a truncating mutation in *chd8*, the sole ortholog of *CHD8* in zebrafish. The *chd8* ^*sa19827*^ mutant line carries a truncating mutation in the first coding exon at position c.C667T (p.Glu223*). First, we determined whether the obtained *chd8* mutant line recapitulates the morphant phenotypes we have previously observed in zebrafish transient knockdown experiments, that is, macrocephaly and a decreased number of enteric neurons (Bernier et al, 2014; Sugathan et al, 2014). Using our established readouts (Golzio et al, 2012; Niederriter et al, 2013; Loviglio et al, 2017), we confirmed the presence of macrocephaly by measuring the distance between the eyes of WT and mutant zebrafish larvae at 5 dpf (Fig S1A). We observed a significant increase in head size in heterozygous *chd8* ^*sa19827/+*^ (mean = 141.7 μm), compared with control *chd8*^*+/+*^ larvae (mean = 133.1 μm) (*t* test, *P* < 0.0001) (Fig S1B). In addition to macrocephaly, we also confirmed that the number of enteric neurons is reduced in the *chd8* ^*sa19827*^ mutant line. HuC/HuD immunostaining on WT and mutant larvae at 5 dpf showed a significant decrease in the number of enteric neurons in the heterozygous *chd8* ^*sa19827/+*^ (mean = 184.6 cells) and homozygous *chd8* ^*sa19827/sa19827*^ (mean = 156 cells) larvae, compared with control *chd8*^*+/+*^ (mean = 242.3 cells) larvae (*t* test, *P* < 0.0001) (Fig S1C and D). We confirmed that enteric neurons localize and distribute properly in both mutant and WT intestines at larval and adult stages as shown by HuC/HuD immunostaining on intestinal cryosections (Fig S1E–H).

### Fewer vagal NCCs emigrate from the neural tube in *chd8* heterozygous mutant embryos

In zebrafish, the ENS, composed of neurons and glial cells, derives mainly from the vagal neural crest (Olden et al, 2008). The observation of a decreased number of mature enteric neurons prompted us to ask whether the initial pool of the vagal NCC was affected in the absence of *chd8*. We used the *Tg2*(*phox2bb:EGFP*) reporter line that marks all vagal NCCs, including migrating enteric NCCs, and immature and differentiated enteric neurons (Taylor et al, 2016; Roy-Carson et al, 2017).

**Figure 1.**
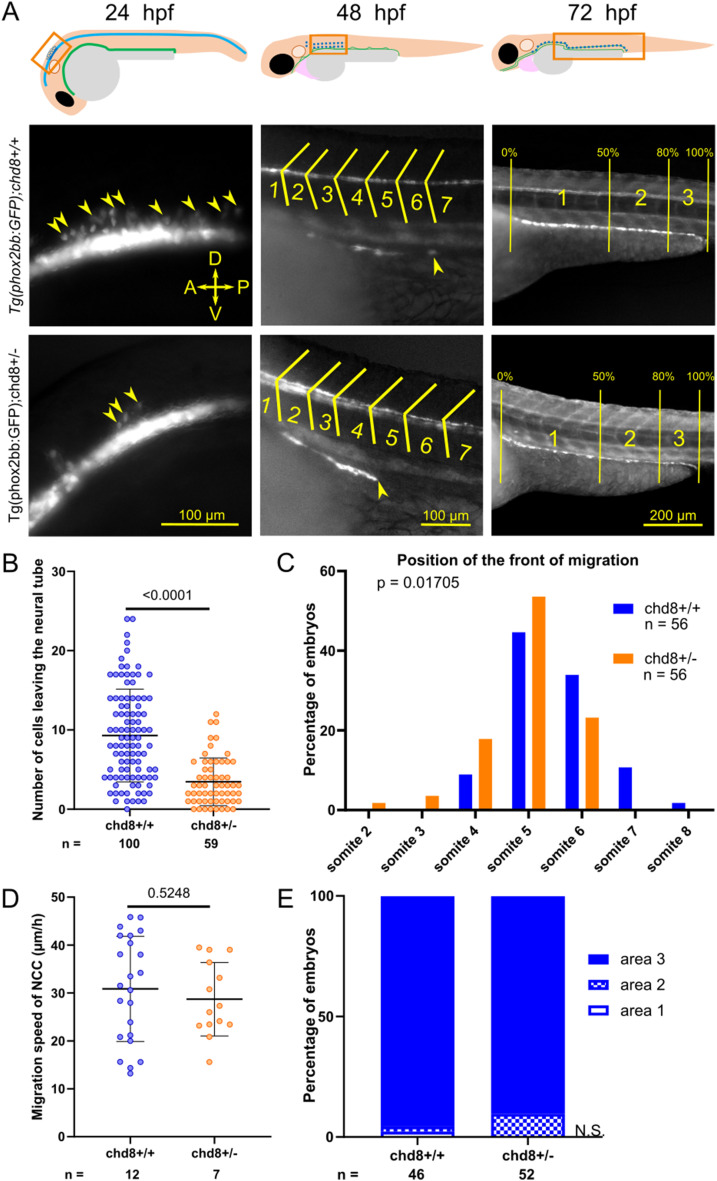
Heterozygous loss of *chd8* leads to induction and early migration defects of vagal NCCs. **(A)** Representative lateral images of *Tg2*(*phox2bb:E*GFP);*chd8*^*+/+*^ and *Tg2(phox2bb:EGFP);chd8*^*sa19827/+*^ zebrafish larvae at 24, 48, and 72 hours post-fertilization (hpf) and schematics showing the imaged areas. At 48 hpf, the somite chevrons are outlined in yellow and the front of migration is indicated by a yellow arrowhead. At 72 hpf, vertical yellow lines delimit the borders of the three following areas: area 1 represents 0–50% migration, area 2 represents 51–80% migration, and area 3 represents 81–100% migration. **(B)** Dot plot of the number of phox2bb-positive cells leaving the neural tube at 24 hpf. A Mann–Whitney test was conducted between pairs of conditions. **(C)** Histogram of the position of the front of migration of enteric NCCs at 48 hpf, using somites as morphological landmarks. Fisher’s exact test was conducted between pairs of conditions. **(D)** Dot plot of the measured speed of enteric NCCs between 50 and 54 hpf for each condition tested. A *t* test was conducted between pairs of conditions. **(E)** Bar graph representing qualitative scoring of the position of the front of migration of enteric NCCs at 72 hpf. Fisher’s exact test was conducted between pairs of conditions. A, anterior; P, posterior; D, dorsal; V, ventral; n, number of embryos or larvae; and n.s., non-significant.

We scored the number of vagal NCCs emigrating from the neural tube in both *chd8* heterozygous mutant and control conditions at 24 hpf (Fig 1A). We observed a significant decrease in the number of NCCs released from the neural tube in *chd8* ^*sa19827/+*^ embryos (mean = 3.458 *phox2bb*+ cells) compared with *chd8*^*+/+*^ embryos (mean = 9.3 *phox2bb*+ cells) (Mann–Whitney’s test, *P* < 0.0001) (Fig 1B). We then followed the migration of the enteric NCCs at several time points. At 48 hpf, we determined the position of the front of migration using the somites as morphological landmarks (Fig 1A). We observed that the position of the front of migration in *chd8*^*sa19827/+*^ embryos was more rostral (between the second and the sixth somite), compared with *chd8*^*+/+*^ embryos (between the fourth and the eighth somite) (Fisher’s exact test, *P* = 0.01705) (Fig 1C). To monitor the migration speed of enteric NCCs at later stages, we took time-lapse images of *Tg2*(*phox2bb:EGFP*); *chd8*^*+/+*^ and *Tg2*(*phox2bb:EGFP*); *chd8* ^*sa19827*/+^ embryos, every 10 min, between 50 and 54 hpf. We did not observe any significant difference in the migration speed of vagal NCCs between *chd8* heterozygous mutant (mean = 28.70 μm/h) and control (mean = 30.85 μm/h) conditions (*t* test, *P* = 0.5248) (Fig 1D). Consistently, we did not observe any significant difference in the position of the front of migration between *chd8*^*sa19827/+*^ and *chd8*^*+/+*^ embryos at 55 hpf (between the 8^th^ and the 14^th^ somite) (Fisher’s exact test, *P* = 0.1803) (Fig S1I and J), 60 hpf (between the 10^th^ and the 14^th^ somite) (Fisher’s exact test, *P* = 0.175) (Fig S1K), and 65 hpf (between the 12^th^ and the 14^th^ somite) (Fisher’s exact test, *P* = 0.599) (Fig S1L). Finally, NCCs from both *chd8* heterozygous mutant and control conditions reached the distal end of the posterior intestine at 72 hpf (Fisher’s exact test, *P* = 0.1515) (Fig 1E), which indicated that the migration capability of vagal NCCs at later stages is not affected when *chd8* expression is reduced.

The migration and proliferation of NCCs are two tightly linked mechanisms (Simpson et al, 2007). We thus assessed whether the proliferation of NCCs was altered in *chd8* heterozygous mutant larvae compared with control larvae. We performed an immunostaining against phospho-histone H3, an M-phase marker, on *Tg2*(*phox2bb:EGFP*) larvae at 4 dpf (Fig S1M). We observed a small but significant increase in the number of proliferative NCCs in *chd8*^*sa19827/+*^ larvae (median = 2), compared with *chd8*^*+/+*^ larvae (median = 1) (Mann–Whitney’s test, *P* = 0.0226) (Fig S1N).

Taken together, our results suggested that key steps of the NCC development, specifically induction and early migration, are affected when *chd8* expression is diminished. Of note, although the induction is finished at 4 pdf, a significant down-regulation of *msx1a*, necessary for NCC induction (Monsoro-Burq et al, 2005), was detected, in enteric NCCs from *chd8* heterozygous mutant larvae at 4 dpf (Supplemental Data 1, log_2_FC = −6.87, *P* = 3.31 × 10^−09^). We also observed a down-regulation of *phox2ba*, one of the two zebrafish orthologs for *PHOX2B*, a gene involved in the migration and survival of enteric NCCs (Pattyn et al, 1999) (Supplemental Data 1, log_2_FC = −5.02, *P* = 0,00019). Our data suggested that the reduced pool of vagal NCCs emigrating from the neural tube is likely the cause of the reduced number of mature enteric neurons observed at later stages.

Supplemental Data 1. Differential expression, DAVID, and PANTHER analyses for all detected differentially expressed genes from RNA-sequencing data. Differential expression analysis was carried out using DESeq2.

### Transcriptional consequences of *chd8* heterozygous loss in enteric neurons

**Figure 2.**
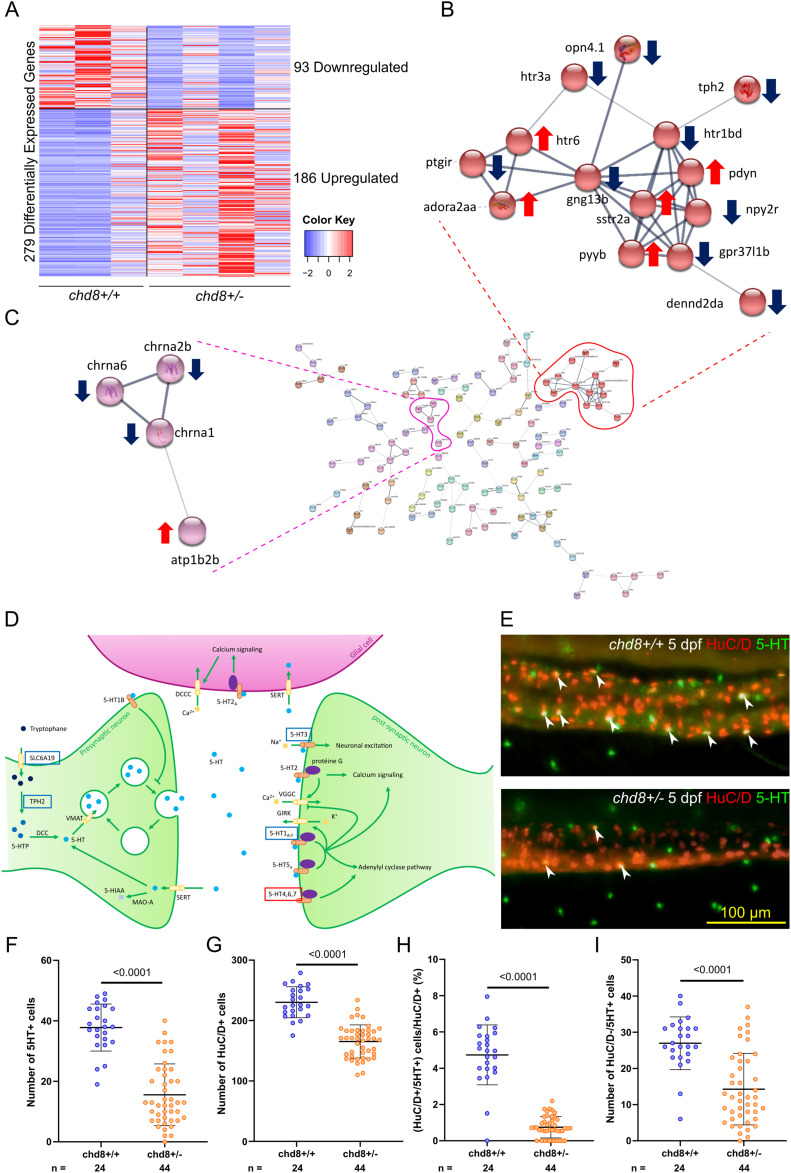
Acetylcholine and serotonin signaling pathways are altered in the enteric neurons of *chd8* ^*sa19827/+*^ larvae. **(A)** Heatmap shows gene expression for the 279 differentially expressed genes: 93 down-regulated genes and 186 up-regulated genes in *chd8* ^*sa19827/+*^. Values have been centered and scaled for each row. Each row represents a single gene. The full list of genes, *P*-values, and associated annotations is provided in Supplemental Data 1. **(B, C)** Protein–protein interaction network of the differentially expressed genes in *chd8* ^*sa19827/+*^. Nodes with no interactions with other proteins of the protein–protein interaction network are not shown. Line thickness indicates the strength of data support. The full network is shown in Fig S2. **(B)** Cluster of 14 proteins including four proteins of the serotonin signaling pathway: *htr1bd*, *htr3a*, *htr6*, and *tph2*. **(C)** Cluster of four proteins including three proteins involved in the acetylcholine signaling pathway: *chrna1*, *chrna2b*, and *chrna6*. **(D)** Serotonergic synapse adapted from KEGG pathways. Genes boxed in blue denote down-regulated genes, and genes boxed in red denote up-regulated genes. **(E)** Representative lateral images of the intestine of *chd8*^*+/+*^ and *chd8* ^*sa19827/+*^ zebrafish larvae at 5 days post-fertilization stained with anti-HuC/D and anti-5-HT monoclonal antibodies to visualize the enteric post-mitotic neurons and the enteric serotonergic cells, respectively. White arrowheads show serotonergic neurons (HuC/D- and 5-HT–positive cells). **(F)** Dot plot of the number of 5-HT–positive cells for each condition tested. A *t* test was conducted between pairs of conditions. **(G)** Dot plot of the number of HuC/D-positive cells for each condition tested. A Mann–Whitney test was conducted between pairs of conditions. **(H)** Dot plot showing the percentage of serotonergic neurons, for each condition tested. A Mann–Whitney test was conducted between pairs of conditions. **(I)** Dot plot of the number of HuC/D-negative/5-HT–positive cells for each condition tested. A Mann–Whitney test was conducted between pairs of conditions. n, number of larvae.

We sorted *phox2bb*-positive neurons from the intestines of *chd8* heterozygous mutant larvae and controls at 4 dpf, and we generated ∼344 million reads by RNA sequencing to monitor changes in genome-wide expression. We performed a differential expression analysis. Overall, 279 genes were differentially expressed (DE) as a consequence of *chd8* suppression (|log_2_(FC)| > 1 and FDR = 0.05). More genes were up-regulated than down-regulated (186 versus 93) (Fig 2A and Supplemental Data 1). Of note, although the enrichment was not significant, we found 74 genes whose human orthologs are associated with Mendelian disorders referenced in the Online Mendelian Inheritance in Man database and 14 DE genes whose human orthologs are associated with autism and referenced in the Simons Foundation Autism Research Initiative database (Supplemental Data 1).

Gene ontology (GO) term enrichment analysis revealed that the GO term “excitatory extracellular ligand-gated ion channel activity” was significantly enriched among the down-regulated genes (*P* = 2.50 × 10^−03^) (Supplemental Data 1). Moreover, the DAVID functional annotation tool showed a significant enrichment of genes involved in the “acetylcholine-gated channel complex” and in “acetylcholine binding” (adjusted *P* = 0.011 and adjusted *P* = 0.041, respectively) among the down-regulated genes. Although not significantly enriched, we also noted that 80 DE genes encode “integral component of membrane” and that 13 DE genes are part of the KEGG signaling pathway “neuroactive ligand–receptor interaction” (Supplemental Data 1). We did not observe any significant enrichment among the up-regulated genes (Supplemental Data 1).

**Figure S2.**
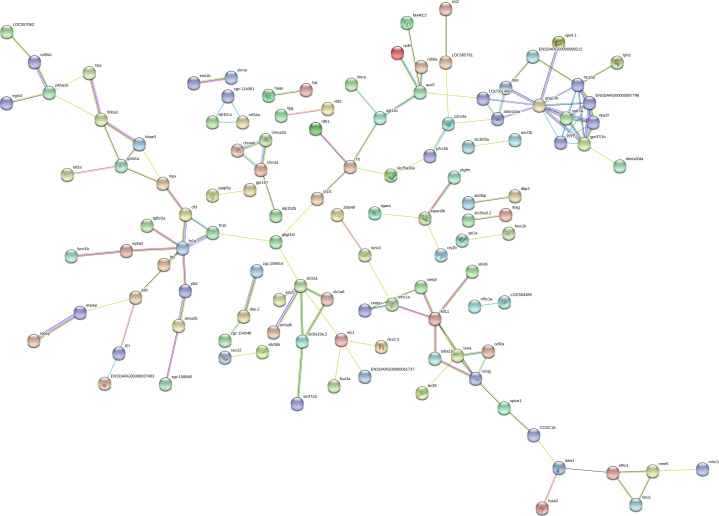
High-resolution image of the protein–protein interaction network of the differentially expressed genes in *chd8* ^*sa19827/+*^. Nodes with no interactions with other proteins of the PPI network are not shown. Markov Cluster Algorithm clustering was performed, using a 3.1 inflation parameter. Red line: fusion evidence; blue line: co-occurrence evidence; yellow line: text mining evidence; green line: neighborhood evidence; purple line: experimental evidence; light blue line: database evidence; and black line: co-expression evidence.

Our transcriptomic data indicated that the expression of several genes directly involved in serotonin metabolism (down-regulated genes: *slc6a19a*.2, *tph2*, *htr1d*, and *htr3a*; up-regulated genes: *htr6* and *aox5*) is altered in *chd8* ^*sa19827*/+^ enteric neurons (Fig 2B–D and Supplemental Data 1) (Kanehisa & Goto, 2000; Walther & Bader, 2003; Seow et al, 2004; Kanehisa, 2019; Kanehisa et al, 2021). We performed STRING analysis on the full list of DE genes, and we generated a full network of the query proteins. The resulting protein–protein interaction (PPI) network had significantly more nodes than expected (*P* = 1.36 × 10^−7^), which indicated that *chd8*-regulated genes are biologically connected (Figs 2B and C and S2). We therefore clustered the genes involved in the PPI network. We found a cluster of 14 genes (down-regulated genes: *opn4*.*1*, *npy2r*, *gpr37l1b*, *dennd2da*, *ptgir*, *gng13b*, *tph2*, *htr1d*, and *htr3a*; up-regulated genes: *pdyn*, *sstr2a*, *pyyb*, *adora2aa*, and *htr6*), including four components of the serotonin signaling pathway (*tph2*, *htr1bd*, *htr3a*, and *htr6*) (Fig 2B), and a cluster of four genes, which included three acetylcholine nicotine receptors (down-regulated genes: *chrna1*, *chrna2b*, and *chrna6*) (Fig 2C).

We then evaluated whether these transcriptomic findings translate into a possible loss or gain of serotonergic cells in the intestine. To visualize the serotonergic neurons and the non-neuronal serotonin-secreting cells, we performed a double immunostaining against HuC/D and serotonin (5-HT) (Njagi et al, 2010; Roach et al, 2013) on *chd8* ^*sa19827/+*^ and control *chd8*^*+/+*^ larvae at 5 dpf (Fig 2E). We observed a significantly decreased number of serotonergic cells in *chd8* ^*sa19827/+*^ larvae compared with controls (mean = 15.55 versus 37.79 5-HT–positive cells) (Mann–Whitney’s test, *P* > 0.0001) (Fig 2F). Because the number of HuC/D-positive neurons is different between *chd8* heterozygous mutants and controls (mean = 165.3 cells versus 230.3 cells; *t* test, *P* > 0.0001) (Fig 2G), we determined the percentage of neurons expressing 5-HT by dividing the number of HuC/D-positive/5-HT–positive cells by the total number of HuC/D-positive cells in both heterozygous mutant and control conditions. In the controls, the serotonergic neurons represented 4.7% of the total number of neurons, whereas in the *chd8* heterozygous mutants, we found only 0.7465% of serotonergic neurons (Mann–Whitney’s test, *P* < 0.0001) (Fig 2H). Moreover, the number of 5-HT–positive cells that are not neurons (HuC/D-negative cells) was also reduced in *chd8* mutants compared with controls (mean = 14.27 versus 26.96 HuC/D-negative/5-HT–positive cells), indicating that the number of serotonin-producing enterochromaffin cells was also reduced (Mann–Whitney’s test, *P* < 0.0001) (Fig 2I).

### The heterozygous loss of *chd8* alters the morphology of the mid- and posterior intestines

**Figure 3.**
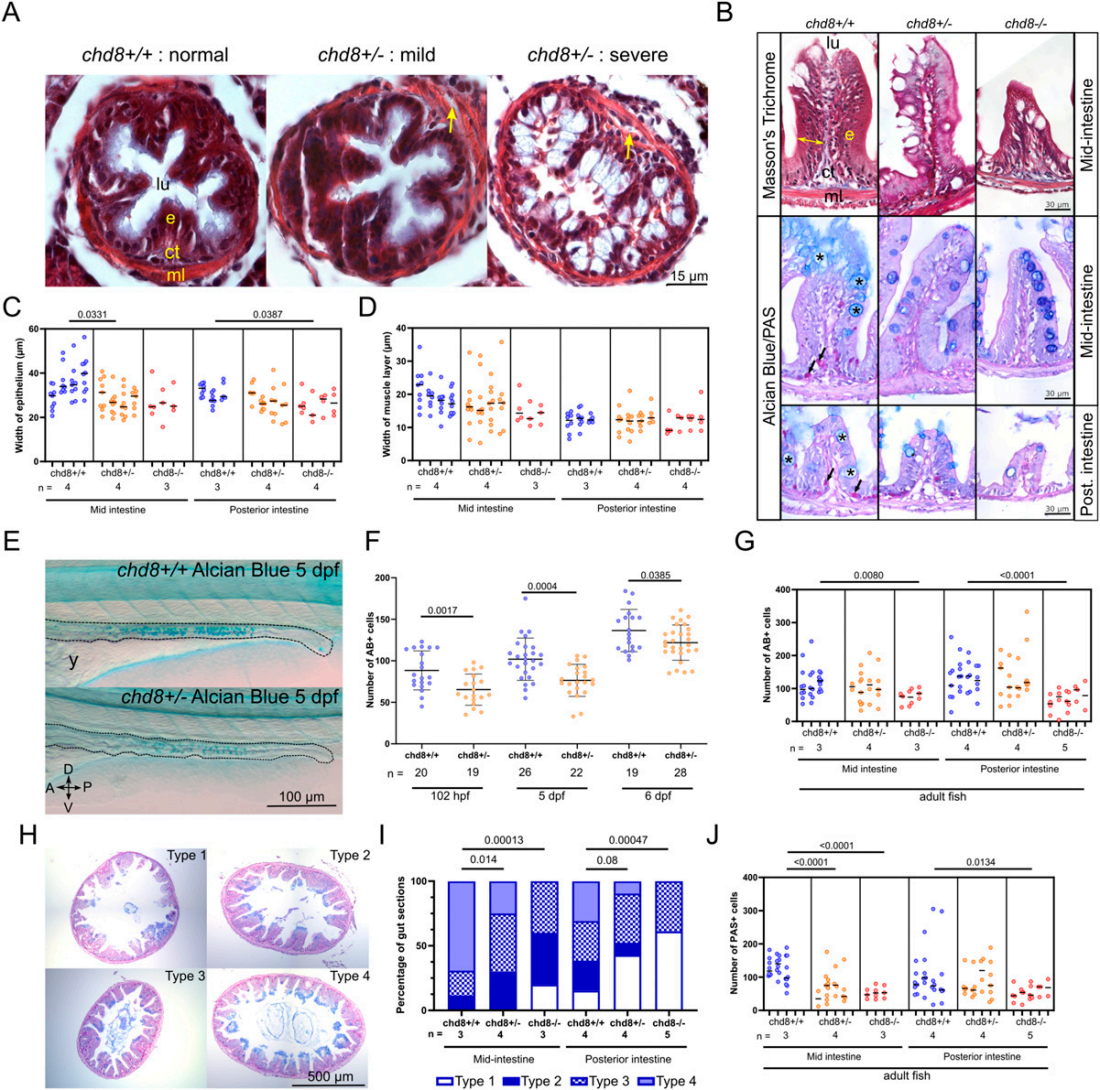
Altered intestinal architecture in *chd8* heterozygous and homozygous mutants at larval and adult stages. **(A)** Representative images of intestinal cross sections of *chd8*^*+/+*^ and *chd8* ^*sa19827/+*^ zebrafish larvae at 5 days post-fertilization (dpf) that underwent Masson’s trichrome staining. Yellow arrows point to gaps between the epithelium and the muscle layers. **(B)** Representative images of cross sections of the mid- and posterior intestines of *chd8*^*+/+*^, *chd8* ^*sa19827/+*^, and *chd8* ^*sa19827/sa19827*^ adult zebrafish that underwent Masson’s trichrome and Alcian blue (AB)/periodic acid–Schiff (PAS) stainings. Black arrows point to PAS-positive cells, and black asterisks indicate AB-positive cells. **(C)** Dot plot of the measured width of the epithelium in the mid- and posterior intestines for each condition tested. **(B)** Width of the epithelium is shown by the yellow double arrowheads in (B). A nested *t* test was conducted between pairs of conditions. **(D)** Dot plot of the measured width of the muscle layers in the mid- and posterior intestines for each condition tested. A nested *t* test was conducted between pairs of conditions. **(E)** Representative lateral images of *chd8*^*+/+*^ and *chd8* ^*sa19827/+*^ zebrafish larvae at 5 dpf that underwent whole-mount AB staining. Dashed lines denote the intestinal margins. **(F)** Dot plot showing the number of AB-positive cells in the intestines of *chd8*^*+/+*^ and *chd8* ^*sa19827/+*^ zebrafish larvae at 5 dpf. A *t* test was conducted between pairs of conditions. **(B, G)** Dot plot showing the number of AB-positive cells, shown by black asterisks in (B), in the mid- and posterior intestines for each condition tested. A nested *t* test was conducted between pairs of conditions. **(H)** Representative images of intestinal cross sections of the mid-intestines of *chd8*^*+/+*^, *chd8* ^*sa19827/+*^, and *chd8* ^*sa19827/sa19827*^ adult zebrafish, stained with AB/PAS. The presence of mucus was scored based on four qualitative types: absence of mucus (type 1), mucus only present at the top border of the villi (type 2), presence of mucus in the intestinal lumen (type 3), and mucus present at the top of the villi and in the intestinal lumen (type 4). **(H, I)** Qualitative scoring of the presence of mucus in mid- and posterior intestines for each condition tested based on the types defined in (H). Fisher’s exact test was conducted. **(B, J)** Dot plot showing the number of PAS-positive cells, shown by black arrows in (B), in the mid- and posterior intestines for each condition tested. A nested *t* test was conducted between pairs of conditions. Each column in panels (C, D, G, J) corresponds to one fish, and each dot represents one tissue section (either an average of the five measurements for panel (C, D) or an absolute number of AB- and PAS-positive cells for panels (G, J), respectively). lu, lumen; e, epithelium; ct, conjunctive tissue; ml, muscle layers; A, anterior; P, posterior; D, dorsal; V, ventral; n, number of adult fish or larvae; and y, yolk.

To investigate further the consequences of *chd8* loss, we evaluated the integrity of the intestine both at larval and at adult stages. To this aim, we performed histological stainings (i.e., Masson’s trichrome and Alcian blue/periodic acid–Schiff’s base reagent [AB-PAS]) on intestinal cross sections (Fig 3A and B). We focused on the mid- and posterior adult zebrafish intestines that resemble the mammalian ileum and colon, respectively (Ng et al, 2005; Wallace et al, 2005).

At the larval stage, we observed a disorganized intestine in 73% of the heterozygous mutant *chd8* larvae compared with only 14% of the *chd8*^+/+^ larvae. A total of 40% of the heterozygous mutant larvae presented with abnormal epithelial layer, abnormal muscle layers, gaps between epithelium and muscle layers, and gaps within the muscle layers (i.e., mild phenotype) and 33% of the mutant larvae exhibited a severe phenotype with a complete absence of intestinal folds, abnormal epithelial cell shape, gaps between epithelium and muscle layers, and gaps within the muscle layers (Fig 3A). The abnormal architecture of the mutant intestines persisted at the adult stage (Fig 3B). We performed Masson’s trichrome staining, and we observed a significant reduction in the epithelium thickness in *chd8* ^*sa19827*/+^ condition compared with controls in the mid-intestine (nested *t* test, *P* = 0.0331) and in *chd8* ^*sa19827*/*sa19827*^ condition compared with controls in the posterior intestine (nested *t* test, *P* = 0.0387) (Fig 3C). The width of the muscle layers was normal in heterozygous and homozygous mutants in the mid-intestine (Fig 3D).

Then, we scored the number of goblet cells in larval intestines at 102 hpf (Ng et al, 2005), 5 dpf, and 6 dpf by performing whole-mount AB staining (Fig 3E). We observed a significant decrease in the number of AB-positive cells in the intestine of *chd8*^*sa19827/+*^ larvae compared with controls (Fig 3F). At the adult stage, the number of mature goblet cells (AB-positive cells, indicated by black asterisks in Fig 3B) was also significantly decreased in both the mid- and posterior intestines of homozygous mutants (nested *t* test, *P* = 0.0080 and *P* < 0.0001, respectively) (Fig 3G). Because the number of mucus-producing goblet cells was reduced, we further scored the presence of mucus on adult intestinal sections and defined four classes: absence of mucus (type 1), presence of mucus on the villi (type 2), presence of mucus in the lumen (type 3), and presence of mucus on the villi and in the lumen (type 4) (Fig 3H). Strikingly, we observed a significant decrease in the presence of the mucus on the villi and in the lumen in heterozygous and homozygous mutants in the mid-intestine (Fisher’s exact test, *P* = 0.014 and *P* = 0.00013, respectively) and in homozygous mutants in the posterior intestine (Fisher’s exact test, *P* = 0.00047) (Fig 3I).

The eosinophils reside in the intestine and exert homeostatic functions, including the maintenance of the protective mucosal barrier that contributes to gut-associated immunity (Jung et al, 2015). The number of PAS-positive eosinophils, indicated by black arrows in Fig 3B, was significantly reduced for both heterozygous and homozygous mutant conditions, compared with controls, in the mid-intestine (nested *t* test, *P* < 0.0001 and *P* < 0.0001, respectively). The number of eosinophils was also reduced for the homozygous mutant condition in the posterior intestine (nested *t* test, *P* = 0.0134) (Fig 3J).

### Loss of *chd8* leads to a perturbed immune balance in the intestine

**Figure 4.**
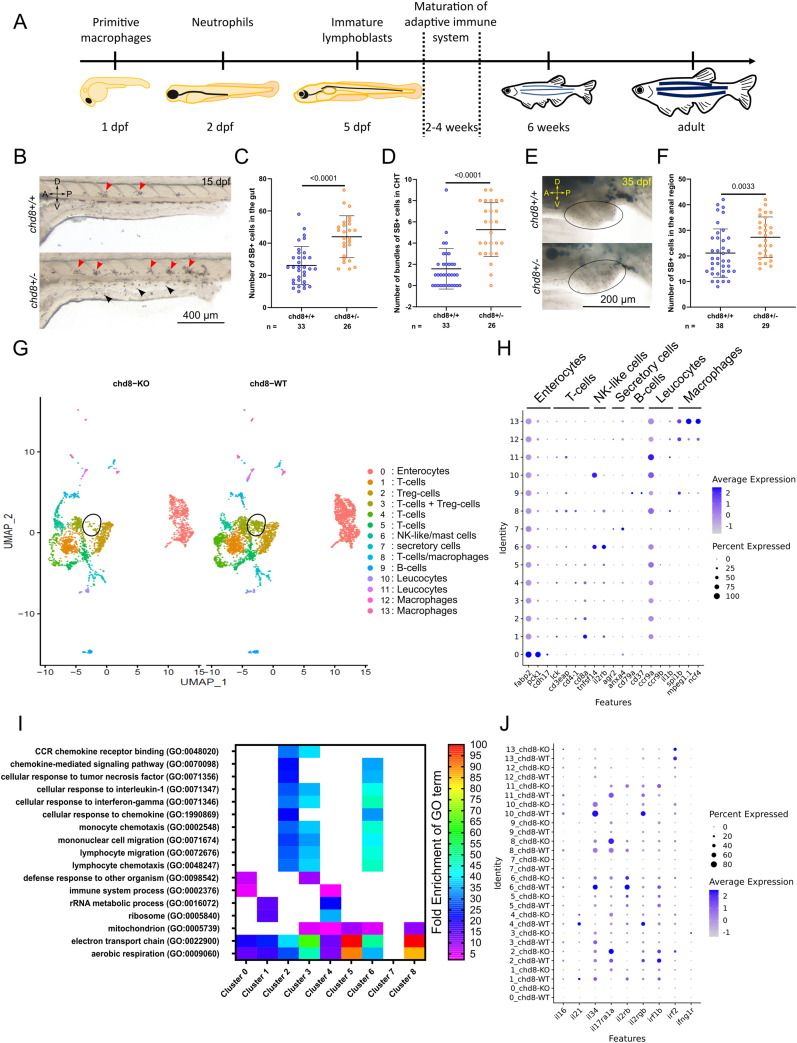
Altered immune balance in the absence of *chd8*. **(A)** Schematic showing the development of the zebrafish innate and adaptive immune system. **(B)** Representative lateral images of *chd8*^*+/+*^ and *chd8*^*sa19827/+*^ zebrafish larvae at 15 days post-fertilization, stained with Sudan Black (SB). Red arrowheads denote the presence of SB-positive bundles (i.e., >five SB-positive cells) in the caudal hematopoietic tissues, and black arrowheads denote the presence of SB-positive neutrophils. **(C)** Dot plot showing the number of SB-positive cells in the intestine for each condition tested. A *t* test was conducted between pairs of conditions. **(D)** Dot plot showing the number of bundles of SB-positive cells in the caudal hematopoietic tissues for each condition tested. A Mann–Whitney test was conducted between pairs of conditions. **(E)** Representative lateral images of the anal region, circled in black, of *chd8*^*+/+*^ and *chd8*^*sa19827/+*^ zebrafish juveniles at 35 days post-fertilization, stained with SB. Large SB-positive areas outside the anal region are lipids and are not quantified. **(F)** Dot plot showing the number of SB-positive cells in the anal region for each condition tested. A Mann–Whitney test was conducted between pairs of conditions. **(G)** UMAP of cells from whole mid- and posterior intestines from both adult homozygous *chd8* mutants and controls, colored by cluster assignment. The black circle denotes *foxp3a*-positive Treg cells. **(H)** Cell-type signatures. The color of the dot shows the level of gene expression, and the size of the dot shows the percentage of cells per cluster that express the gene of interest. **(I)** Heatmap showing a subset of statistically significant GO terms, represented by at least 40 genes, identified using a PANTHER overrepresentation test (FDR = 0.05) for up-regulated genes in eight clusters. The colors represent the fold enrichment for each GO term. The full list of associated terms and *P*-values for each cluster is provided in Supplemental Data 2. **(J)** Dysregulation of immune-related genes. The color of the dot shows the level of the expression of genes of interest, and the size of the dot shows the percentage of cells per cluster that express the gene of interest. A, anterior; P, posterior; D, dorsal; V, ventral; and n, number of adult fish or juveniles.

We hypothesized that intestinal architecture changes, including the thinning of the epithelium and muscle layers, the decreased numbers of goblet cells and eosinophils, and a decreased amount of produced mucus, could be accompanied by a perturbed immune balance in the intestine. In zebrafish, the innate immune system is functional with mature neutrophils at 2 dpf (Le Guyader et al, 2008). Adaptive immunity, in the form of mature B and T cells, appears between week 2 and week 4 (Fig 4A) (Lam et al, 2004; Page et al, 2013).

We used Sudan Black B (SB), which is a lipophilic dye that integrates into granule membranes and therefore marks mature, granulated neutrophils. We observed a significant increase in the number of neutrophils, indicated by black arrowheads, in the intestinal tissue (*t* test, *P* < 0.0001) in mutant larvae at 15 dpf (Fig 4B and C). We also noticed the presence of SB-positive cell bundles, indicated by red arrowheads, that abut the caudal artery dorsally and the somite muscle limit ventrally, consistent with previous reports (Walters et al, 2010). Although these SB-positive cell bundles in the caudal hematopoietic tissue normally disappear between 7 and 13 dpf in WT larvae (Walters et al, 2010), we still observed a significantly high number of these SB-positive cell bundles (Fig 4B) in heterozygous mutants compared with controls at 15 dpf (Mann–Whitney’s test, *P* < 0.0001) (Fig 4D). A modest but significant increase in the number of neutrophils is also observed in heterozygous juvenile mutants compared with juvenile controls in the anal region of the posterior intestine at 35 dpf (Mann–Whitney’s test, *P* = 0.0033) (Fig 4E and F).

Our data indicated that the numbers of eosinophils and neutrophils, two mediators of innate immunity, are changed in the absence of *chd8*. To investigate further the impact of *chd8* loss on adaptive intestinal immunity, we collected the mid- and posterior intestines of controls and homozygous mutant adult males and we performed single-cell transcriptomic analyses using 10× Genomics technology. We analyzed a total of 6,339 cells: 3,865 cells for control and 2,474 for homozygous mutant conditions.

**Figure S3.**
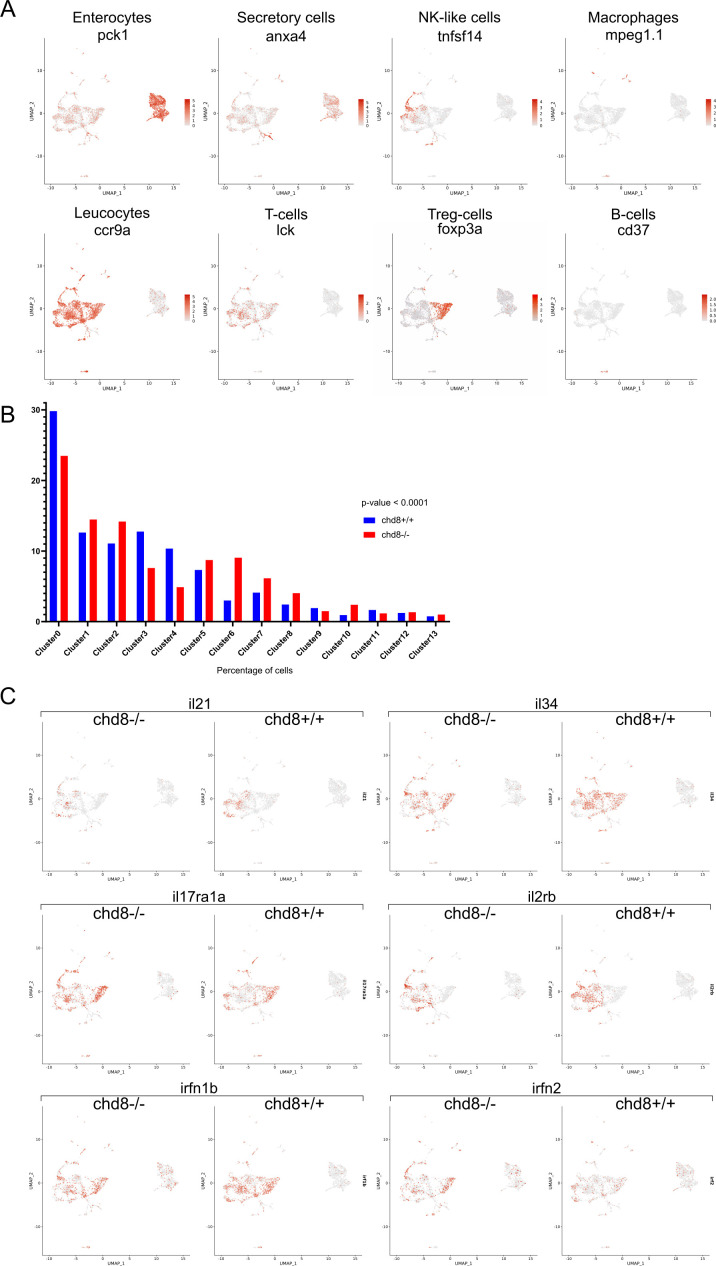
Expression levels of cell-type markers and interleukins. **(A)** UMAP. The color shows the level of the expression of *pck1*, *anxa4*, *tnfsf14*, *mpeg1*.*1*, *ccr9a*, *lck*, *foxp3a*, and *cd37*, markers of enterocytes, secretory cells, NK-like cells, macrophages, leukocytes, T cells, Treg cells, and B cells, respectively. **(B)** Repartition of cells in the clusters. Fisher’s exact test was conducted between pairs of conditions. **(C)** UMAP. The color shows the level of the expression of *il21*, *il34*, *il17ra1a*, *il2rb*, *irfn1b*, and *irfn2*.

Using the Seurat R package, 14 cell clusters were identified (Fig 4G). To determine cell cluster identity, we used known sets of markers (Carmona et al, 2017; Masud et al, 2017; Gu et al, 2019). For instance, we used enterocyte markers such as *fabp2*, *pck1*, and *cdh17*. T cells were identified by *lck*, *cd3eap*, *cd4*-*1*, and *cd8a*. The expression of *tnfsf14* and *il2rb* defined the NK-like cell cluster, and *cd79a* and *cd37* are expressed in B cells. We used *ccr9a*, *ccr9b*, and *il1b* as leukocytic markers. Last, macrophages were identified by *spi1b*, *mpeg1*.*1*, and *ncf4* (Figs 4H and S3).

We first analyzed the clusters by comparing the repartition of the cells in the clusters in homozygous mutant and control conditions. We observed a significant difference in the overall repartition of cells in the clusters between *chd8*^*sa19827/sa19827*^ homozygous mutants and *chd8*^*+/+*^ controls (Fisher’s exact test, *P* = 5.52 × 10^−54^). Strikingly, we found that the population of T-regulatory lymphocytes expressing *foxp3a* in cluster 3 is almost absent in the homozygous mutant condition (Figs 4G and S3).

GO term enrichment analysis on DE genes between *chd8* homozygous mutants and controls in each cluster revealed that several GO terms associated with innate immune response and inflammation were significantly enriched in *chd8* homozygous mutants (Fig 4I and Supplemental Data 2). In particular, the GO terms “lymphocyte chemotaxis” (GO:0048247), “lymphocyte migration” (GO:0072676), “mononuclear cell migration” (GO:0071674), “monocyte chemotaxis” (GO:0002548), “cellular response to interferon-gamma” (GO:0071346), and “cellular response to interleukin-1” (GO:0071347) were significantly enriched among the up-regulated genes in T cells and NK-like cell clusters (clusters 2, 3, and 6). Furthermore, the GO terms “chemokine-mediated signaling pathway” (GO:0070098), “cellular response to tumor necrosis factor” (GO:0071356), and “cellular response to chemokine” (GO:1990869) were also enriched among the up-regulated genes in T cells and NK-like cell clusters (clusters 2 and 6). Mitochondria play a part in the regulation of inflammation (Collins et al, 2004; Bahat et al, 2021). Consistently, we observed that the GO terms “electron transport chain” (GO:0022900) and “aerobic respiration” (GO:0009060) are enriched among the up-regulated genes in enterocytes and T-cell clusters (clusters 0, 1, 2, 3, 4, 5, 6, and 8). Furthermore, the GO term “mitochondrion” (GO:0005739) is enriched among the up-regulated genes in T-cell clusters (clusters 3, 4, 5, 6, and 8), and among the down-regulated genes in enterocyte cluster (cluster 0).

Supplemental Data 2. PANTHER analyses on differentially expressed genes from single-cell RNA-seq data. For each cluster, salmon color shows the PANTHER analysis performed on up-regulated genes and blue color shows the PANTHER analysis performed on down-regulated genes.

Interleukins and interferon signaling pathways are instrumental in the activation of the immune response (Germolec et al, 2018). Thus, we asked whether interleukins, interleukin receptors, and interferons are DE between homozygous mutants and controls (Figs 4J and S3). Strikingly, we found that three interleukins were significantly down-regulated among the T-cell clusters: the pro-inflammatory *il16* was down-regulated in a T-cell cluster (cluster 3), whereas *il21* and the pro-inflammatory *il34* were significantly down-regulated in T-cell cluster 1 and in T-cell clusters 1 and 6, respectively. In addition, the expression of three interleukin receptors was altered in several T-cell clusters. The receptor for the pro-inflammatory cytokine il17a, *il17ra1a*, was up-regulated in clusters 1 and 2, whereas the receptors for il2, *il2rb* and *il2rgb*, were down-regulated in clusters 1 and 4, respectively. The interferon signaling pathway was also affected in the homozygous mutants. Specifically, *irf1b* was up-regulated in enterocytes (cluster 0), *irf2* was up-regulated in T cells (cluster 2), and the interferon-γ ortholog *ifng1r* was up-regulated in T cells (cluster 3).

Taken together, our data strongly suggested that both innate immunity and adaptive immunity are activated, possibly due to mucosal barrier breakdown, which ultimately leads to intestinal inflammation when *chd8* expression is diminished.

## Discussion

GI problems in ASD-associated neurodevelopmental syndromes are common; however, their etiology remains largely unknown. Here, we investigated the role of autism-associated *chd8* during the enteric NCC development and in the maintenance of gut homeostasis. Using zebrafish, we showed that *chd8* acts quite early during the NCC development and that the reduction of its expression affects the number of enteric NCCs emigrating from the neural tube and their early migration. In mature enteric neurons, *chd8* indirectly or directly regulates serotonin and acetylcholine signaling pathways. Moreover, we found that the numbers of both serotonergic neurons and enterochromaffin cells were reduced in the intestine, indicating that *chd8* is essential during the differentiation of enteric NCCs into serotonergic neurons and that its diminished expression likely leads to hyposerotonemia in the intestine. Finally, we identified a role of *chd8* in the maintenance of gut homeostasis. In both juvenile and adult zebrafish mutants, the tissue examination revealed a compromised intestinal architecture accompanied by an accumulation of neutrophils and the decreased numbers of goblet cells and eosinophils in the intestine. Single-cell sequencing of the whole intestine confirmed a global disruption of the immune balance in the intestine, with exacerbated immune response and a drastic reduction in the anti-inflammatory regulatory T cells.

### ASD-associated GI complaints: are they neurocristopathies?

Although *CHD8* disruption is associated with GI complaints (Bernier et al, 2014), its function during the vagal NCC development has never been examined. Here, we showed that the reduction of the expression of *chd8* affects several steps of the vagal NCC development including induction, early migration, and differentiation into enteric neurons. We found a decreased number of vagal NCCs emerging from the neural tube at 24 hpf, suggesting a perturbed induction when *chd8* is inactivated. This possibility is further supported by our transcriptomic data showing that *msx1a*, necessary for NCC induction (Monsoro-Burq et al, 2005), was down-regulated in enteric NCCs from mutant larvae. We thus propose that *chd8* plays a role in the induction of the vagal neural crest, by regulating, directly or indirectly, the factors of induction. Early intervention of *chd8* may be important for the newly delaminated vagal NCC progenitors to proceed to migratory stages. This possibility is in line with recent transcriptomic work on cranial NCCs in mice showing that the complex Chd8/Twist1 controls delaminatory and early migratory markers (Fan et al, 2021). Contrary to Hirschsprung’s disease (HSCR; MIM#142623), a congenital condition associated with a failure of vagal NCCs to colonize the intestine (Okamoto & Ueda, 1967; Edery et al, 1994), we found that the reduced expression of *chd8* does not prevent the completion of the rostro-caudal colonization of the GI tract by vagal NCCs. The absence of aganglionic segments in the posterior intestine of *chd8* mutants further suggests that reduction of *chd8* expression do not affect drastically the initial NCC–progenitor pool. Our work shows that the etiology of motility disturbances in patients with *CHD8* mutations is, in part, due to the impaired NCC development but is rather different from neurocristopathies affecting the GI tract such as HSCR.

### Loss of *chd8* leads to hyposerotonemia in the intestine

The NCC differentiation is governed by a precise sequence of fate decisions at the right time and place (Mayor & Theveneau, 2013). We and others have shown that *chd8* regulates gene expression in pathways involved in neurodevelopment, supporting a role for chromatin remodelers in neuronal differentiation (Sugathan et al, 2014; Durak et al, 2016; Sood et al, 2020; Fan et al, 2021). However, *chd8* function in enteric neurons has never been reported. Therefore, we examined the role of *chd8* by establishing the functional genomic effects in enteric mature neurons after reducing its expression to a level comparable to that expected from the heterozygous inactivating mutations found in ASD (Bernier et al, 2014). Hence, in the heterozygous mutant condition, we observed fewer enteric neurons that exhibited dysregulated cholinergic and serotonergic signaling pathways in mid- and posterior intestines.

Acetylcholine is the most common neurotransmitter to induce GI smooth muscle contractions (Bolton, 1979). We found that three genes coding subunits for nicotinic acetylcholine receptors, *chrna1*, *chrna2b*, and *chrna6*, are down-regulated in enteric neurons in the absence of *chd8*. Mutations in *CHRNA1* and *CHRNA6* have been implicated in fast-channel congenital myasthenic syndrome (MIM#608930) characterized by early-onset progressive muscle weakness and chronic pain (Wieskopf et al, 2015; Natera-de Benito et al, 2017). Of note, a decrease in cholinergic signaling in individuals with duplication of *CHRFAM7A*, that encodes a dominant negative α7-nAChR inhibitor, is associated with IBD (Baird et al, 2016; Rueda Ruzafa et al, 2021). We propose that the absence of *chd8* might reduce cholinergic signaling in the intestine, which could, in turn, affect contraction capability and alter intestinal transit.

In the nervous system, serotonin (5-HT) is produced either by 2–3% of enteric neurons by the tryptophan hydroxylase 2 (TPH2) or by the enterochromaffin cells via TPH1 (Côté et al, 2003; Walther & Bader, 2003; Spohn & Mawe, 2017). Conventional functions of serotonin in the gut involve intrinsic reflexes, including stimulation of propulsive motility patterns, epithelial secretion, and vasodilation (Mawe & Hoffman, 2013). We found an altered expression of several receptors for serotonin in neurons with reduced *chd8* expression including *htr3a*, *htr6*, and *htr1d*. The 5-HT_3_ receptor is known to be involved in intestinal motility (Mawe & Hoffman, 2013), whereas the 5-HT_6_ and 5-HT_1_ receptors regulate the adenylyl cyclase signaling pathway, which, in turn, regulate the hyperexcitability of neurons (Zhong et al, 1992; Zhong & Wu, 2004). We also found that both *slc6a19a*.*2*, coding a carrier involved in the absorption of tryptophan, the precursor of serotonin, and the enzyme *tph2* are down-regulated in mutant larvae, which indicates that serotonin is likely underproduced by the enteric neurons when the expression of *chd8* is reduced. In addition, the numbers of both 5-HT–positive neurons and 5-HT–producing enterochromaffin cells were decreased in the heterozygous mutant intestines. Our work suggests that *chd8* tightly controls the serotonin pathway in both neuronal and non-neuronal 5-HT–positive cells. Notably, changes in the number of intestinal enterochromaffin cells and in serotonin production have been observed in patients with IBD, and in animal models of colitis (El-Salhy et al, 1997; Coates et al, 2004) and in a zebrafish knockout model for *shank3a/b* (James et al, 2019). Moreover, people with IBD who experience constipation often have lower plasmatic levels of serotonin (Atkinson et al, 2006). Recent work using *Drosophila melanogaster* indicates that the loss of *CHD8*/*CHD7* ortholog, *kismet*, leads to increased levels of serotonin in the brain and in the proventriculus and the anterior midgut, which can be zebrafish equivalents of the intestinal bulb and the anterior part of the mid-intestine, respectively (Coll-Tané et al, 2021). Our work is in contradiction with this study regarding observed levels of serotonin in the mid-intestine. Here, using a vertebrate model, our data suggested that the loss of *chd8* likely leads to hyposerotonemia in the mid- and posterior intestines.

### Consequence of *chd8* loss on mucosal barrier maintenance

The *chd8* adult mutants exhibited a compromised intestinal architecture. Notably, we observed the thinning of the intestinal epithelium layer, a reduced number of goblet cells accompanied by the reduced presence of mucus in the intestinal lumen, and the decreased levels of eosinophils. Altogether, these perturbations likely alter the structure and protective functions of the mucosal barrier. This possibility is further supported by the observed increased number of neutrophils in the intestine of mutant larvae as early as 15 dpf. It is known that in the case of mucosal injury, inflammatory monocytes are recruited into the mucosal wound site after neutrophil infiltration to facilitate the recovery of the mucosal barrier (Xue & Falcon, 2019). The mucosal barrier is constituted by antimicrobial peptides and mucus layer constructed by intestinal epithelial cells. Recently, it has been shown that intestinal mucus layer maintenance depends on eosinophil presence in the lamina propria because eosinophil-deficient mice had significantly the decreased numbers of mucus-secreting goblet cells in the small intestine (Jung et al, 2015). Moreover, *muc2*-deficient mice, in which the mucus layer is defective, develop spontaneous colitis (Van der Sluis et al, 2006). A decreased mucosal barrier function and neutrophil infiltration are observed in the intestines of patients with IBD (Jäger et al, 2013). Although further research is needed to determine whether *chd8* is necessary for the establishment and/or the maintenance of the mucosal barrier, we speculate that patients with *chd8* mutations are more prone to bacterial infection and/or colitis because of the altered mucosal barrier.

### Immune balance is perturbed in the absence of *chd8*

To combat bacterial antigens, intestinal epithelial cells indirectly or directly interact with innate and adaptive immune cells by presenting antigens to dendritic cells or T cells, or by expressing cytokines, chemokines, hormones, and enzymes (Hoytema van Konijnenburg et al, 2017; Allaire et al, 2018). Our single-cell transcriptomic data revealed a strong impact on immune cell clusters when *chd8* is absent. Strikingly, we found that the population of *foxp3a*-positive regulatory T cells (Treg) is reduced in the intestine of adult *chd8* mutants. In addition, we observed a significant enrichment for GO terms related to the innate immune response such as response to interferon-γ, cellular response to chemokines, lymphocyte, and monocyte chemotaxis, and cellular response to tumor necrosis factor in T-cell clusters, suggesting an overly active immune response in the intestine when *chd8* is absent. Furthermore, we found that the expression of *il17ra1a*, the receptor for IL-17, is increased in mutants compared with controls. IL-17–producing Th17 lymphocytes and Treg cells represent two arms of an immune response (reviewed in Lee [2018]). The balance between Th17 and Treg cells is critical for the health of the host. Th17 cells participate in the defense against extracellular bacterial and fungal infections. On the contrary, Treg cells regulate the immune response and maintain immune homeostasis. An excessive activation of Th17 leads to inflammation and autoimmune disease. Of note, an increased Th17/Treg ratio is associated with a higher severity of the autistic traits in children with ASD (Moaaz et al, 2019). Our findings strongly suggest that *chd8* loss leads to a perturbed Th17/Treg balance, which provokes an excessive inflammatory response in the intestine.

Taken together, we propose a model in which the reduced expression of *chd8* induces the breakdown of the mucosal barrier, which, in turn, drives intestinal vulnerability to infection. As a consequence, the intestine is challenged by bacterial antigens, and innate immune response is activated. Inflammation is subsequently maintained in challenged *chd8* mutant intestines because of a reduced number of Treg cells and increased IL-17 signaling through its receptor IL-17RA.

Several limitations exist in the present study. First, because we used a constitutive knockout *chd8* zebrafish line, it is rather difficult to establish cause–effect relationships, especially concerning the cell-autonomous or non-autonomous effect of *chd8* on the NCC induction, migration, and differentiation. However, some of our findings are in favor of co-occurring developmental defects because of the pleiotropic effects of *chd8*. Second, several studies report that individuals with ASD harbor altered gut microbiota (Tomova et al, 2015; Lim et al, 2017). Although unlikely to be the disease driver (Yap et al, 2021), it will be of interest to investigate whether the microbiota is affected in the absence of *chd8*. Third, we postulated that the depleted pool of Treg cells might be unable to restrain IL-17 signaling, which leads to persistent and uncontrolled inflammation. However, further studies are necessary to examine, specifically, the activity of the Th17 lymphocytes and whether downstream effectors of IL-17RA are activated when *chd8* is absent.

Our work aimed to unveil the intricacies of GI complaints in autism. Although some mechanisms remain to be elucidated, our work provides several lines of evidence, suggesting that GI complaints in individuals with *CHD8* mutations are due to complex interplay between neuronal, epithelial, and immune cells. In the future, it will be essential to pursue the unraveling of the links between the ENS development, mucosal barrier, and immune balance and to characterize precisely the etiology of the GI complaints in specific ASD population to determine therapeutic actions.

## Materials and Methods

### Zebrafish husbandry

Zebrafish (*Danio rerio*) were raised and maintained as described in Westerfield (2000). Adult zebrafish were raised in 15-liter tanks containing a maximum of 24 individuals, and under a 14-h:10-h light–dark cycle. The water had a temperature of 28.5°C and a conductivity of 200 μS and was continuously renewed. The fish were fed three times a day, with dry food and *Artemia salina* larvae. Embryos were raised in E3 medium, at 28.5°C, under constant darkness. The AB strain obtained from the European Zebrafish Resource Center was used as WT for this study. The mutant line *chd8* ^*sa19827*^, carrying the mutation c.C667T (p.Glu223*), was obtained from the European Zebrafish Resource Center (#24433; EZRC), and the w37Tg transgenic line, carrying the construct *Tg2*(*phox2bb:EGFP*), was obtained from the International Resource Centre for Zebrafish (#ZL1748; ZIRC). All fish lines reproduce normally, and *chd8* homozygote mutants were recovered in the expected Mendelian ratio. A skewed sex ratio was observed with almost only males. Experiments on adult zebrafish were performed using 1-yr-old males. The developmental stages of zebrafish embryos and larvae are indicated in the text and figures. For zebrafish embryos and larvae, both males and females were used because the sex can only be determined at 2 mo of age. All animal experiments were carried out according to the guidelines of the Ethics Committee of IGBMC, and ethical approval was obtained from the French Ministry of Higher Education and Research under the number APAFIS#15025-2018041616344504.

### Genotyping of the *chd8* ^*sa19827*^ mutant line

Adult fish were anesthetized in 80 μg/ml tricaine. Fin clips were digested in 50 μl of 50mM NaOH for 15 min at 95°C, and the reaction was neutralized by adding 5 μl of 1 M Tris–HCl, pH 7. The genomic region encompassing the sa19827 mutation was amplified by PCR, using the following primers: 5′-GTCAGACTCAAGTGCTGCAG-3′ and 5′-GACACTTTGGTCGGAT-3′. The PCR product was digested by the *Rsa*I enzyme, a restriction enzyme whose restriction site is disrupted by the sa19827 mutation. We ran the digestion product on a 2% agarose gel for 30 min at 135 V. For control *chd8*^*+/+*^, two bands are detected (250 and 180 base pairs); for heterozygous *chd8* ^*sa19827*/+^, three bands are detected (428, 250, and 182 base pairs); and for homozygous *chd8* ^*sa19827*/^ ^*sa19827*^, a single 428 base pair band is detected. In figures, *chd8*^+/−^ refers to heterozygous *chd8* ^*sa19827*/+^ and *chd8*^−/−^ refers to homozygous *chd8* ^*sa19827*/^ ^*sa19827*^.

### Imaging of enteric NCCs in the intestine

Transgenic *Tg2*(*phox2bb:EGFP*) larvae were imaged at 24, 48, and 72 hpf, on a lateral view, in PBS–Tween 0.1%, using a MacroFluo ORCA Flash macroscope (Leica). At least 15 larvae were imaged per condition, and z-stacks were acquired. We used the ImageJ software to create a “maximum intensity” projection. To monitor the migration speed of enteric NCCs, we took time-lapse pictures of *Tg2*(*phox2bb:EGFP*); *chd8*^*+/+*^ and *Tg2*(*phox2bb:EGFP*); *chd8* ^*sa19827/+*^ embryos, every 10 min, between 50 and 54 hpf, using a time-lapse video microscope (Zeiss). The migration speed was assessed by measuring the distance traveled by the front of migration for 1 h, and two measurements were taken per embryos, on two consecutive hours.

### Immunostainings on zebrafish larvae

Zebrafish larvae were fixed in 4% PFA for 1–3 h, then incubated for 10 min in PBS–Triton 0.5%, and washed three times in PBS–Triton 0.1% for 30 min, at room temperature. The larvae were then incubated in blocking solution (PBS–Triton 1%/DMSO 1%/BSA 1%/FBS 1%) for 1 h at room temperature, then incubated in primary antibody diluted in the blocking solution, overnight, at room temperature. The next day, the larvae were rinsed three times in PBS–Triton 0.1% for 30 min at room temperature and incubated in secondary antibody diluted in the blocking solution, for 2 h at room temperature, in the dark. The larvae were stored in PBS, at 4°C, in the dark. A complete list of primary and secondary antibodies is available in the Key Resources Table. The larvae were imaged, on a lateral view, in PBS-Tween 0.1%, using a MacroFluo ORCA Flash macroscope (Leica). At least 15 larvae were imaged per condition, and z-stacks were acquired. We used the ImageJ software to generate a “maximum intensity” projection and scored the number of fluorescent cells using the ICTN 1.6. plug-in.

### Flow cytometry and RNA sequencing

**Figure S4.**
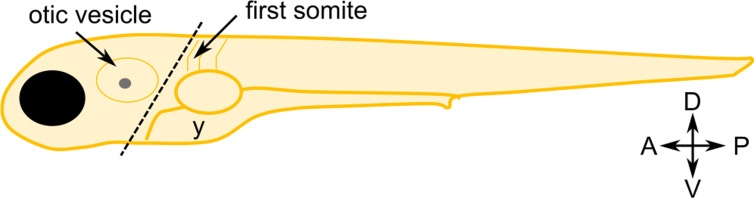
Schematic showing the axial level where the head was removed for bulk RNA sequencing. The heads were removed as depicted by the dashed line. A, anterior; P, posterior; D, dorsal; V, ventral; and y, yolk.

*chd8*^*+/+*^ and *chd8* ^*sa19827/sa19827*^ males were crossed with *Tg2*(*phox2bb:EGFP*) females, and the eggs were incubated at 28.5°C. At 4 dpf, the larvae were euthanized in 2 mg/ml tricaine diluted in RPMI and the heads of the larvae were discarded. The heads were removed at the level of the first somite, as shown in Fig S4. Of note, *phox2bb* is also expressed in spinal cord neurons; the sorted GFP-positive cells include NCCs and spinal cord neurons. The rest of the larval bodies were collected in a 2-ml Eppendorf tube; all RPMI was removed and replaced with 1 ml of Trypsin–EDTA 1× (ref 59417C-100ML; Sigma-Aldrich). The digestion was stopped after 10 min by adding 50 μl of inactivated fetal calf serum. The tubes were centrifuged at 2000*g*, during 2 min at room temperature, the supernatant was removed, and 100 μl of FACS Max medium was added (ref T200100; AMSBIO). The larval bodies were then placed on a cell filter (diameter 40 μm, ref 141378C; Dutscher), previously moistened with 100 μl of FACS Max medium, and the cells were filtered, using a 1-ml syringe plunger. The filter was rinsed with 400 μl of FACS Max medium; the cells were collected and placed in a 1.5-ml Eppendorf tube. The GFP-positive cells were immediately sorted, using an ARIA Fusion cell sorter and an excitation wavelength of 488 nm. We stored the GFP-positive cells at −80°C, in 10 μl of PBS-RNAsin 1 U/μl. Each biological replicate consists of 950–1,300 cells harvested from 80 larvae from independent clutches. Harvesting of the GFP-positive cells was conducted on four different days; we thus controlled for batch differences when performing the subsequent differential gene expression analysis. Full-length cDNAs were generated using the Clontech SMART-Seq v4 Ultra Low Input RNA Kit for Sequencing (Takara Bio Europe), according to the manufacturer’s instructions with 12 cycles of PCR for cDNA amplification by SeqAmp polymerase. 600 pg of pre-amplified cDNA was then used as input for Tn5 transposon tagmentation by the Nextera XT DNA Library Preparation kit (96 samples) (Illumina) followed by 12 cycles of library amplification. After purification with Agencourt AMPure XP beads (Beckman Coulter), the size and concentration of libraries were assessed by capillary electrophoresis. Libraries were then sequenced on an Illumina Hiseq4000 sequencer as single-end 50-bp reads. The reads were preprocessed with cutadapt version 1.10 (Martin, 2011) and mapped on the zebrafish genome (GRCz11 assembly), using the STAR software version 2.5.3a (Dobin et al, 2013). For each sample, more than 85% of the preprocessed reads were uniquely mapped and could be used to quantify gene expression using htseq-count, version 0.6.1p1 (Anders et al, 2015), with annotations from Ensembl version 98. One of the *chd8*^*+/+*^ samples was excluded from the analysis because the number of reads aligned on *chd8* locus was very low, unlike in the other *chd8*^*+/+*^ samples. The differential gene expression analysis between enteric neurons of *chd8*^*+/+*^ and *chd8* ^*sa19827/+*^ larvae, controlling for batch differences, was conducted using the DESeq2 Bioconductor package version 1.16.1 (Love et al, 2014) (Wald’s test and *P*-value adjustment using the Benjamini and Hochberg method [Benjamini & Hochberg, 1995]).

We conducted a GO analysis on the list of up-regulated and down-regulated genes, and on the full list of DE genes, using a PANTHER overrepresentation test (using the website geneontology.org). We also used the DAVID functional annotation tool (version 6.8) on the same lists of genes. Finally, we performed STRING analysis on the full list of DE genes and we generated a full network of the query proteins, using all active interaction sources and a minimum interaction score of 0.4. We then clustered the genes involved in the PPI network, using the MCL clustering method and an inflation parameter of 3.1. We generated the heatmap using the Galaxy tool heatmap.2: toolshed.g2.bx.psu.edu/repos/iuc/ggplot2_heatmap2/ggplot2_heatmap2/3.0.1. The data were neither transformed nor clustered, and it was scaled by row.

### Paraffin sections and histological stainings

*chd8*^*+/+*^, *chd8* ^*sa19827/+*^, and *chd8* ^*sa19827/sa19827*^ male adult zebrafish were euthanized in 800 μg/ml tricaine solution. Mid- and posterior intestines per condition were collected (Wallace et al, 2005) and then fixed in 10% neutral buffered formalin for 3 h at room temperature. They were rinsed twice in 1× PBS and twice in 70% ethanol. The intestines were paraffin-embedded according to the standard procedure. Paraffin blocks were cut at a thickness of 5 μm with a Leica RM2235 manual rotary microtome. Masson’s trichrome stain was performed as follows: tissues were post-fixed in Bouin’s solution during 1 h at 56°C and rinsed abundantly in running water for 7 min. Sections were stained in Weigert’s hematoxylin (C.I.75290; Sigma-Aldrich) for 10 min. After a wash in water, sections were stained in Biebrich scarlet-acid fuchsin solution for 2 min. After another wash in water, slides were differentiated in a phosphotungstic acid solution for 15 min and directly transferred to aniline blue solution (C.I.42755; Sigma-Aldrich) for 30 min. AB/PAS stain was conducted according to the standard procedure with a Harris hematoxylin (C.I.75290; Sigma-Aldrich) counterstain. All the stained tissue sections were cleared with a Histosol-clearing agent, mounted with Eukitt medium, and imaged with a motorized Leica DM4000B microscope equipped with a CoolSnap CF Color camera (Photometrics), 10×/0.30 (objective), 100×/1.30 oil (objective). Illumination was done with a halogen lamp 100 W. The images were merged with the Navigator interface driven by LasX software.

### Analyses of intestinal sections

The counts and measurements were done manually with the Fiji software. For the detection of fluorescent labeled cells, batches of embryos or larvae were analyzed using ImageJ with the plug-in ITCN 1.6 to quantify automatically the fluorescent positive cells. All the histological analyses were done blind to the genotypes. For epithelium width measurements (Fig 3B and C), the measurements were done in the lower one-third of the villus as indicated by the double-headed arrow. A total of five villi per tissue section were randomly chosen and measured. For muscle layer measurements (Fig 3B and D), the muscle layer was measured at five random locations on each tissue section. For both epithelium and muscle measurements, a total of 3–10 consecutive tissue sections per fish were analyzed for each intestinal region to reduce sectioning artifacts (mid- and posterior intestine) and a total of three to five adult fish per genotype were analyzed. For AB- and PAS-positive cell scoring, all AB-positive and PAS-positive cells present in each tissue section were counted. A total of 3–10 tissue sections per fish for each intestinal region and a total of three to five adult fish per genotype were analyzed. For HuC/D-positive neuron counting in adult fish, the intestinal section was arbitrarily divided into four identical quadrants (Fig S1G) and all HuC/D-positive neurons were counted on three consecutive sections per fish and plotted as the number of neurons per quadrants. A total of six to seven fish from independent clutches per condition were analyzed.

### AB staining on 5 dpf larvae

The embryos were fixed at 5 dpf, in 4% paraformaldehyde for 24 h at 4°C, and then transferred to 100% methanol and kept at −20°C for at least 24 h. Embryos were rehydrated in decreasing concentrations of methanol in PBS and then washed in PBS. They were bleached for 20–30 min (3% H_2_O_2_/0.5% KOH), then washed in PBS-T. They were incubated in AB (A3157; Sigma-Aldrich) overnight, under agitation. The next day, the embryos were washed throughout the day in acidic ethanol (70% ethanol/5% hydrochloric acid/25% H_2_0). The embryos were then incubated in 100% ethanol for 10 min and then stored in 100% glycerol. The embryos were imaged, on a lateral view, using a stereo microscope Leica MZ125.

### In situ hybridization

We amplified *D*. *rerio phox2bb* transcript by performing PCR using the following primers: 5′-ATTCCTCTGCCTACGAGTCC-3′ and 5′-TAATACGACTCACTATAGGGTGGCTCCGTTCTGTCTTTGT-3′, on cDNA generated from total RNA extracted from 24 hpf embryos as a template. We labeled sense and antisense RNA probes with digoxigenin and performed whole-mount RNA in situ hybridization on 55, 60, and 65 hpf embryos as described in Thisse and Thisse (2008). Minor changes have been applied compared with the original protocol as follows: the larvae were beached for 10 min and treated with proteinase K for 1 min. The larvae were imaged, on a lateral view, using a stereo microscope Leica MZ125.

### Sudan black B staining

*chd8*^*+/+*^ and *chd8* ^*sa19827/+*^ larvae at 14 dpf and juveniles at 35 dpf were fixed in 4% PFA for 4 h at room temperature. They were washed three times for 5 min in 1 ml of 1× PBS, under agitation. They were then incubated in 1 ml of filtered Sudan Black B working solution (0.036% [wt/vol] Sudan Black B [15928; Merck], 0.1% phenol, and 94% ethanol), in tubes covered in aluminum foil at room temperature for 1 h, under agitation. They were then washed 3 times for 5 min in 70% ethanol under agitation and washed in PBS-Tween 0.1%. They were bleached in 1 ml of depigmentation solution (0.1% KOH, 1% H_2_O_2_) for 5 min under agitation. Finally, they were washed twice in 1 ml PBS-Tween 0.1% for 5 min at room temperature under agitation. The larvae and juveniles were imaged, on a lateral view, using a stereo microscope Leica MZ125. A total of five or more SB-positive cells define a bundle.

### Single-cell RNA sequencing

*chd8*^*+/+*^ and homozygous *chd8* ^*sa19827/sa19827*^ male adult zebrafish were euthanized in 800 μg/ml tricaine solution. Three adult fish were dissected per genotype; the guts were harvested and placed in RPMI at room temperature. Three guts were used per condition. The guts were rolled on a paper moistened with RPMI to remove the fat residue, then placed in RPMI with 10% fetal calf serum, and cut into small pieces that were placed in 1 ml of digestion medium (1 ml of RPMI/12 μl of activated fetal calf serum/10 mg of dispase/collagenase) for 15 min, at 37°C, under agitation at 500 rpm (FA-45-24-11). The cells were then filtered on a cell filter (diameter 40 μm, ref 141378C; Dutscher), using the plunger of 1-ml syringe. The cell concentration and viability were assessed with trypan blue. Samples consisted of >90% viable cells and were processed on the Chromium Controller from 10× Genomics. 10,000 total cells were loaded per well. A single-cell 3′ mRNA-seq library was generated according to 10× Genomics User Guide for Chromium Single Cell 3′ Reagent Kits (v3 Chemistry). Briefly, Gel Beads-in-Emulsion were generated by combining barcoded gel beads, a RT master mix containing cells, and a partitioning oil onto Chromium Chip B. After full-length cDNA synthesis and barcoding from polyadenylated mRNA, Gel Beads-in-Emulsion were broken and pooled before cDNA amplification by PCR using 11 cycles. After enzymatic fragmentation and size selection, sequencing libraries were constructed by adding Illumina P5 and P7 primers, and sample index via end repair, A tailing, adaptor ligation, and PCR with 14 cycles. Library quantification and quality control were performed using Bioanalyzer 2100 (Agilent Technologies). Libraries were then sequenced on an Illumina NextSeq550 sequencer (2 runs: 28 + 96 and 101 + 101). Alignment, barcode, and UMI filtering and counting were performed with Cell Ranger 3.1.0 count, using GRCz11 assembly and Ensembl release 98 annotations. The filtered gene-barcode matrix obtained with Cell Ranger count was further analyzed using R 4.0.2 and Seurat 3.2.0 (Stuart et al, 2019). Cells with at least 200 and less than 2,000 expressed genes and with less than 5% of mitochondrial reads and genes expressed in at least three cells were retained for further analysis. After normalization (NormalizeData with the LogNormalize method), the two datasets were integrated (finding anchors using FindIntegrationAnchors and using these anchors to integrate the two datasets with IntegrateData using dimensions 1:50). After scaling the integrated data (ScaleData), we performed a principal component analysis with 50 principal components (RunPCA). We use this PCA as input to perform a Uniform Manifold Approximation and Projection (UMAP) dimensional reduction in order to visualize the datasets (RunUMAP). Cell clustering was performed using FindNeighbors (with the first 50 principal components) and FindClusters (with a resolution of 0.3). To identify marker genes that are conserved between conditions for each cluster, we used FindConservedMarkers. DE genes between homozygous mutants and controls were identified using FindMarkers in each cluster. We conducted a GO analysis on the list of up-regulated and down-regulated genes in each cluster, using a PANTHER overrepresentation test (using the website geneontology.org). Graphical representations were performed using DimPlot (UMAP), DotPlot (dot plots), and FeaturePlot (feature plots, where cells were represented in order of expression).

### Quantification and statistical analyses

We used GraphPad Prism v8.0.2.263 (GraphPad Software) to visualize data. Statistical analyses were performed using either GraphPad Prism v8.0.2.263 or R v4.1.0. All experiments from this study were performed at least on three biological replicates with at least 15 larvae per clutch, from three independent clutches, or at least three adult zebrafish per genotype. Adult zebrafish were raised from three independent clutches. When two groups were compared, the normality of the distribution was assessed by performing a Shapiro–Wilk test. If the distribution was not normal, a Mann–Whitney test was conducted between pairs of conditions. If the distribution was normal, a F test was conducted between pairs of conditions to assess whether the variances could be considered equal. If the variances were not statistically different, a *t* test was conducted between pairs of conditions. If the variances were statistically different, a Welch *t* test was conducted between pairs of conditions. When multiple groups were compared, the Kruskal–Wallis test was conducted. On dot plots, unless otherwise specified, the individual measurements are plotted, and the mean and SD are represented. For qualitative data (e.g., classes based on the presence of mucus), a Fisher exact test was conducted between pairs of conditions to assess whether the distribution of samples in the different categories was significantly different. Two groups were considered statistically different if *P* < 0.05. No data were excluded from analyses, unless otherwise specified in the results.

## Data Availability

### Materials availability

This study did not generate new unique reagents.

### Data and code availability

(1) Single-cell RNA-sequencing data and bulk RNA-sequencing data have been deposited at GEO and are publicly available as of the date of publication. Accession numbers are listed in the Key Resources Table. Microscopy data reported in this study will be shared by the lead contact upon request.
(2) This study does not report the original code.
(3) Any additional information required to reanalyze the data reported in this study is available from the lead contact upon request.

**Table:** 

Resource	Source	Identifier
Antibodies		
Anti-HuC/HuD antibody (mouse), dil 1:200	Invitrogen	A-21271
Anti-5-HT antibody (rabbit), dil 1:500	Sigma-Aldrich	S5545-25UL
Anti-PH3 antibody (rabbit), dil 1:750	Santa Cruz	sc-8656-R
IgG (H-L) mouse Alexa Fluor 594 (goat), dil 1:500	Invitrogen	A-11005
IgG (H-L) mouse Alexa Fluor 488 (goat), dil 1:500	Invitrogen	A-11001
IgG (H-L) rabbit Alexa Fluor 488 (donkey), dil 1:500	Invitrogen	A-21206
Experimental models		
*chd8* ^*sa19827*^ mutant zebrafish line	EZRC	24433
w37Tg transgenic zebrafish line	ZIRC	ZL1748
Deposited data		
RNA sequencing on enteric NCCs at 4 dpf	This study	GSE184359
Single-cell RNA sequencing on the adult intestine	This study	GSE184363

## Supplementary Material

Reviewer comments
